# Optical Responses From Zero Optical‐Path‐Length Object

**DOI:** 10.1002/nap2.70243

**Published:** 2026-08-10

**Authors:** Da Yong Lee, Moongul Byun, Robert Magnusson, Jae Woong Yoon

**Affiliations:** ^1^ Department of Physics Hanyang University Seoul South Korea; ^2^ Department of Electrical Engineering University of Texas Arlington Texas USA

**Keywords:** guided‐mode resonances, optical path length, photonic lattices, two‐dimensional materials

## Abstract

Optical path length (OPL) is a key concept that determines elementary and advanced optical effects from classical optical components to cutting‐edge nanophotonic devices. In this paper, we treat objects with their physical and OPL thickness simultaneously vanishing and show that such objects in principle can produce remarkably strong optical effects under certain conditions associated with dipole‐preserving compression of an ordinary three‐dimensional medium. Such two‐dimensional (2D) sheets essentially produce elementary optical responses of reflection, diffraction, and localization in the same manner as a quantum‐mechanical delta‐function potential‐well problem. On this basis, we propose periodic array structures that enable resonant excitations and versatile spectral features favorable for practical applications taking advantages of their minimally space‐occupying and least inertial embodiment. Importantly, the proposed zero‐OPL‐thickness elements can be effectively created in practice by using strongly polarizing ceramics or 2D materials including graphene and transition‐metal dichalcogenide monolayers.

## Graphical abstract

This work address a fundamental question—what is the ultimately minimal embodiment of optical objects that produce useful optical effects? In fact, such an object does not require nonzero physical and optical‐path‐length volume as opposed to the conventional perception. Two‐dimensional polarizing sheets are the minimal optical objects and practically realizable using existing media such as 2D materials and highly polarizing ceramics.

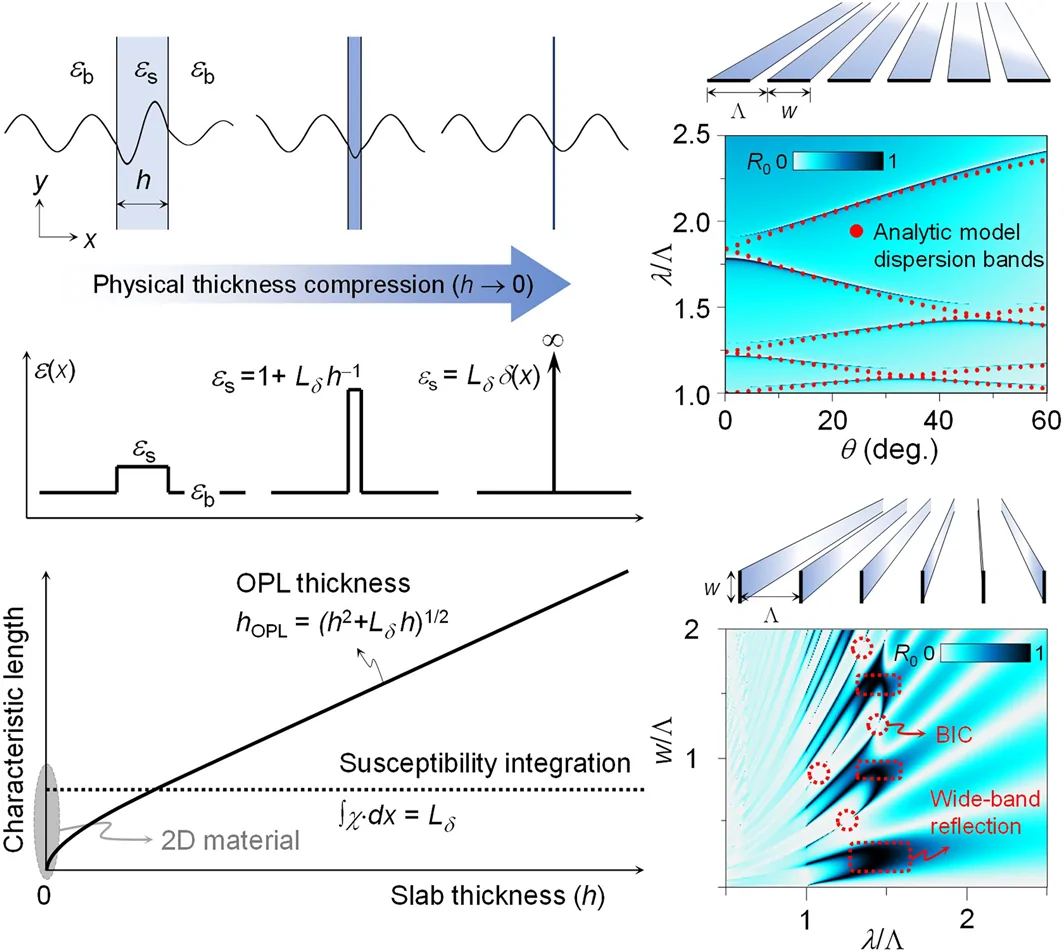

## 1 Introduction

In our traditional wisdom from the optical physics, one may regard that optical effects from a structure tend to vanish in the extreme sparsity limit where optical path length (OPL) and physical thickness of its element size simultaneously approach zero. This statement is generally reasonable because the phase delay of optical fields in the constituent media diminishes in such a limit and so do the subsequent elementary processes by reflection, diffraction, and scattering. In another words, a zero‐OPL‐size element allows no space for optical waves to oscillate within and thereby can be treated as being trivially nothing as far as its optical effects are concerned.

Nevertheless, recent development of novel two‐dimensional (2D) materials and their remarkable optical effects suggest that the nonzero OPL‐size condition may not be a fundamental requirement for producing substantial optical effects. For example, robust waveguiding of visible and infrared optical fields were experimentally demonstrated with a single sheet of atomically thin transition metal dichalcogenide (TMDC) and graphene monolayers [1, 2, 3, 4, 5, 6, 7].

We point out that characteristic OPL‐thickness of such atomic monolayer systems effectually vanishes. In the standard classical‐electrodynamics perspective, one may treat an atomic monolayer as a continuously distributed surface‐dipole sheet and thereby its responses can be described by an electric susceptibility distribution:

(1) $$\chi \left(r\right)=L_{\delta }\delta \left(x\right),$$

where δ(x) is the Dirac delta function and $L_{\delta }$ is a polarizing‐strength coefficient taking the length dimension. When we calculate OPL thickness *h*_OPL_ of the surface‐dipole sheet defined by Equation (1), it is strictly zero because

(2) $$h_{\text{OPL}}=\lim_{h\to 0}\int_{-h/2}^{+h/2}\sqrt{1+L_{\delta }\delta \left(x\right)}\;dx=0.$$

Therefore, it suggests in principle that the conceptually minimal embodiment of an optical element can possibly occupy no OPL volume. This further implies that one may construct extremely thin, minimally space‐occupying, and least inertial optical or electromagnetic‐wave components by utilizing any strongly polarizing sheets which approximate the δ(x) susceptibility.

In this letter, we address this simple but fundamental argument. We show that the zero‐OPL δ(x) susceptibility is indeed the conceptually minimal requirement for producing elementary processes of reflection, diffraction, waveguiding, and various subsequent effects by their combinations. In addition, we further investigate resonant arrays of zero‐OPL δ(x)‐susceptibility sheets. We show that the resonant arrays efficiently produce combined effects of the elementary processes and enable remarkably useful functionalities beyond the previous waveguiding demonstrations. We discuss the implications of our result in 2D‐material science and applications in low‐frequency spectral regions where material species with extremely high polarizability are abundant [8, 9, 10, 11].

## 2 Results

### 2.1 Physical Basis

Developing our fundamental argument in an intuitive manner, we consider a planar slab of a fictitious medium whose dielectric constant is inversely proportional to slab thickness *h* such that

(3) $$\varepsilon_{s}=1+L_{\delta }\;h^{-1},$$

as schematically illustrated in Figure 1A. Note that media behaving like this do not exist in nature but we employ it as a fictitious, imaginary material that conceptually describes physical implication of a dielectric function involving the Dirac *δ* function, as explained hereafter.

**FIGURE 1:**
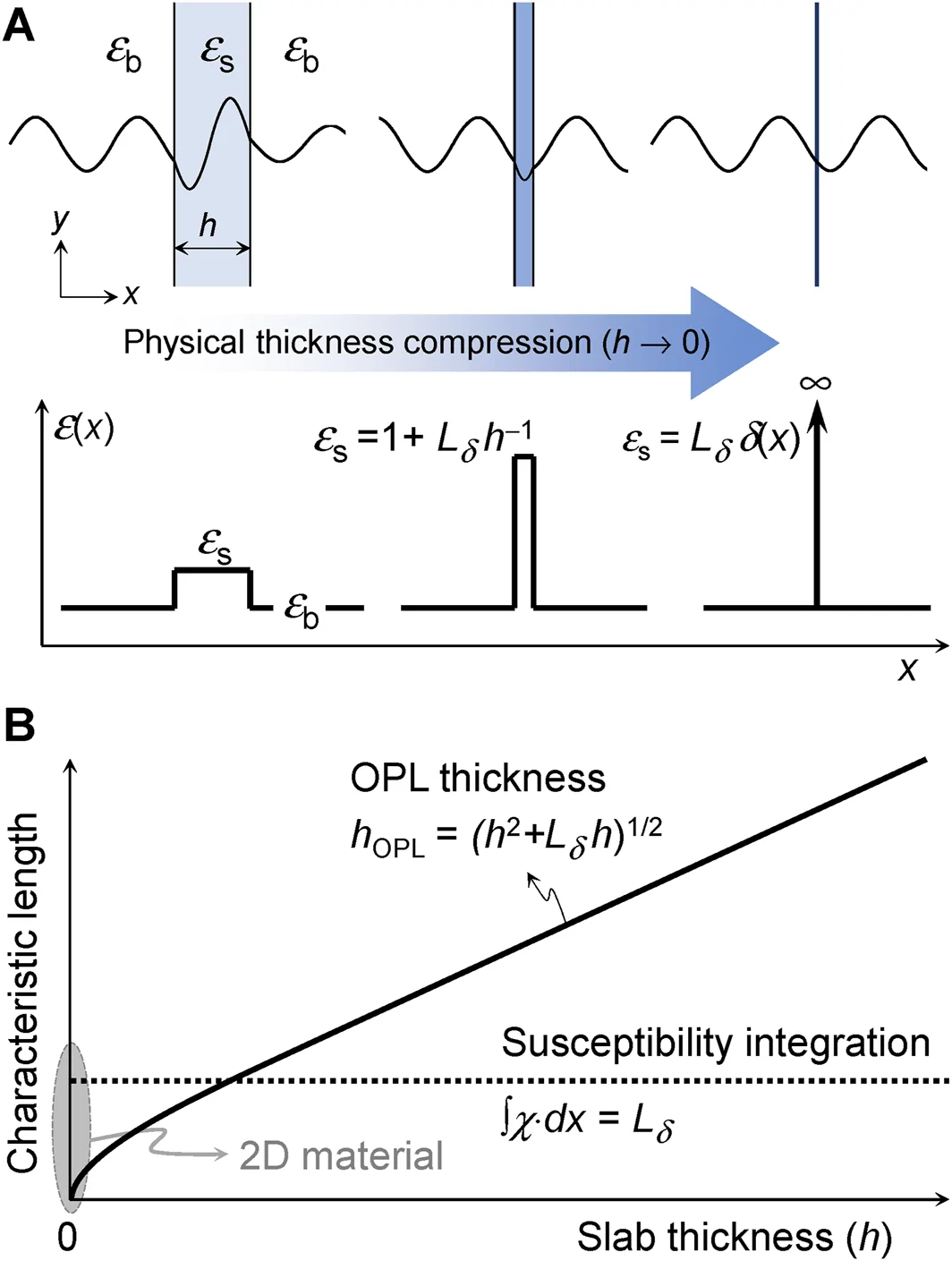
Concept of a zero‐OPL *δ*‐dielectric sheet. (A) Schematic of a fictitious dielectric slab following Equation (3). The slab gradually morphs into a 2D dielectric material in its vanishing thickness limit (*h* → 0). (B) Essential *h*‐dependence of the OPL thickness and susceptibility integration length of the fictitious dielectric slab.

In deep‐subwavelength thickness limit of h≪λ, this slab tends to a 2D material with zero‐OPL thickness. As *h* approaches zero, OPL thickness $h_{\text{OPL}}=\varepsilon_{s}1^{/2}h={\left(h^{2}+L_{\delta }\;h\right)}^{1/2}$ of the slab vanishes, whereas the susceptibility integration over the slab thickness, that is, $\int \chi \cdot \;dx=\int \left(\varepsilon_{s}-1\right)\cdot \;dx=L_{\delta }$, is a nonzero constant at $L_{\delta }$., as shown in Figure 1B. Therefore, this fictitious dielectric slab properly models zero‐OPL 2D dielectric materials as its physical thickness approaches zero, that is, *h* → 0.

Although *h*_OPL_ obviously vanishes in the *h* → 0 limit, one may expect significant optical responses because the surface dipole density of the fictitious slab remains constant at a nonzero value. Surface dipole density *S*_*P*_ under a unit applied electric field is determined by

(4) $$S_{P}=\varepsilon_{0}\int_{-h/2}^{+h/2}\chi \left(x\right)\;dx=\varepsilon_{0}L_{\delta },$$

and this is independent of *h*. Therefore, the fictitious slab by Equation (3) in the vanishing OPL‐thickness limit (*h*_OPL_ → 0) is considered as a medium compression that transforms a 3D medium into a 2D material while preserving the amount of the constituent electric dipoles which are the fundamental sources of optical responses.

More rigorous argument is inferred from mathematical principles of the classical electrodynamics. For an ideal zero‐OPL *δ*‐dielectric sheet by Equation (1), the Maxwell equations lead to a wave equation for frequency‐domain electric field **E** as

(5) $$\left(\nabla^{2}+\varepsilon_{b}{k_{0}}^{2}\right)E=-{k_{0}}^{2}L_{\delta }\delta \left(x\right)\;E_{s},$$

where *ε*_*b*_ is dielectric constant of the background medium, and **E**_*s*_ denotes the local electric field at the sheet surface, that is, **E**_*s*_(*y*, *z*) = **E**(*x* = 0, *y*, *z*). Equation (5) has two major implications. First, the *δ*‐dielectric sheet acts as an ideal impulse source with strength −*k*_0_^2^ *L*_*δ*_ **E**_*s*_ and, thereby, constitutes a mathematically minimal source that perturbs function **E**.

Second, following the standard mathematical procedure for the Poisson equation, the complete field solution for a given incident field can be calculated by imposing two interface conditions:

(6) $$\lim_{\Delta \to 0}E^{∥}\left(x=-\Delta \right)=\lim_{\Delta \to 0}E^{∥}\left(x=+\Delta \right)={E_{s}}^{∥},$$

(7) $$\lim_{\Delta \to 0}\left\{\left[\frac{\partial E^{∥}}{\partial x}\right]\left(x=-\Delta \right)-\left[\frac{\partial E^{∥}}{\partial x}\right]\left(x=+\Delta \right)\right\}={k_{0}}^{2}L_{\delta }\;{E_{s}}^{∥},$$

where superscript “‖” indicates the field component tangential to the sheet. Equation (6) is simply the continuity of tangential field, whereas Equation (7) specifies the amount of discontinuity in the first‐order spatial derivative of the tangential field normally across the sheet. We note that this is in exact analogy of the quantum‐mechanical Dirac‐*δ*‐function potential well problem.

The zero‐OPL *δ*‐dielectric sheet leaves its footprint in the wavefunction in the form of discontinuity in the first‐order derivative as stated in Equation (7). Such footprint produces significant partial reflection. Using Equations (6) and (7), we derive the reflection coefficient *r*_*s*/*p*_ under transverse‐electric (TE, subscript “s”) and transverse‐magnetic (TM, subscript “*p*”) plane wave incidence on a zero‐OPL *δ*‐dielectric sheet as

(8) $$r_{s}=i\;\sin\;u_{s}\;\exp\left(iu_{s}\right),$$

(9) $$r_{p}=-i\;\sin\;u_{p}\;\exp\left(iu_{p}\right),$$

where parameter *u*_*s*/*p*_ is given by

(10) $$u_{s}=\tan^{-1}\left(\sigma_{\delta }\;\sec\;\theta \right)\;\text{and}\;u_{p}=\tan^{-1}\left(\sigma_{\delta }\;\cos\;\theta \right),$$

for a given angle of incidence *θ*. Here, *σ*_*δ*_ = π*ε*_*b*_^−1/2^(*L*_*δ*_/*λ*) is the normalized polarizing‐strength coefficient with *λ* being the wavelength in vacuum. The partial transmission coefficients are subsequently given by *t*_*s*_ = 1 + *r*_*s*_ for TE polarization and *t*_*p*_ = 1 − *r*_*p*_ for TM polarization, respectively. In case of *σ*_*δ*_ = 1 at *θ* = 0 for example, *u*_*s*_ = *u*_*p*_ = 45° and this yields reflectance |*r*_*s*_|^2^ = |*r*_*p*_|^2^ = 50%. In the weakly polarizing limit of $\sigma_{\delta }\ll 1$, the reflectance under normal incidence is approximated to ${\left|r_{s}\right|}^{2}={\left|r_{p}\right|}^{2}\approx {\left|\sigma_{\delta }\right|}^{2}$. Although this value is regarded as being insignificant, it might be large enough to produce strong effects if multiple sheets are in a periodic or appropriately configured array such that strong constructive interference builds up for discrete angular channels or modes in a similar manner as multilayer coatings, diffraction gratings, and holographic optical elements.

Another crucial effect from a zero‐OPL *δ*‐dielectric sheet is optical guiding for TE polarization. Equation (7) results in a fundamental TE guided‐mode solution with an effective index:

(11) $$n_{f}=n_{b}\sqrt{1+{\sigma_{\delta }}^{2}}.$$

The guiding effect is robust because the sheet supports it even in the weakly polarizing limit $\sigma_{\delta }\;<<\;1$ where *n*_eff_ ≈ *n*_*b*_(1 + *σ*_*δ*_^2^/2) > *n*_*b*_.

In contrast, a zero‐OPL *δ*‐dielectric sheet does not support any guided mode for TM polarization. In the deep‐subwavelength thickness limit (h≪λ), the effective index *n*_eff_′ for TM‐polarized fundamental mode tends to

(12) $${n_{f}}^{\text{TM}}\;≃\;n_{b}\sqrt{1+\frac{k_{0}h}{2}},$$

where core dielectric constant is given by Equation (3). Obviously, ${n_{f}}^{\text{TM}}$ → *n*_*b*_ for a zero‐OPL *δ*‐dielectric sheet (*h* → 0). Consequently, a zero‐OPL *δ*‐dielectric sheet does not support any TM‐guided mode.

In addition, the boundary conditions in Equations (6) and (7) predict that the sheet does not respond to any electromagnetic field configurations in which **E**_*s*_ is normal to the sheet and, thereby, the sheet does not leave any trace in the field itself as well as its first‐order derivative distributions. Therefore, efficient polarization‐selective optical or electromagnetic elements might be developed from certain structures based on zero‐OPL *δ*‐dielectric sheets.

In our theoretical argument here, we assume the *local* macroscopic electromagnetism in treating zero‐OPL *δ*‐dielectric sheets. Therefore, our description is valid as far as the relevant in‐plane wave vector remains small compared to the inverse characteristic length scales of the constituent material [12, 13]. Throughout the visible and infrared spectral domains, in‐plane wave vector on the order of the free‐space wave number satisfy this validity condition as being consistent local 2D sheet models in the previous waveguiding demonstrations with graphene and TMDC monolayers [14, 15].

For media hosting exciton resonances that lead to intrinsically nonlocal, long‐range dipole responses [12], however, the rigorous description might require the nonlocal electromagnetism even in the small‐wave‐vector limit. Such resonances appear in narrow bands over the visible and near‐infrared domains. For example, MoS_2_, WSe_2_, and MoSe_2_ monolayers have such exciton resonances at wavelengths of 660 nm, 750 nm, and 790 nm, respectively [16]. The Lorentz oscillator model of electric susceptibility for these intrinsic exciton resonances describes the nonlocal dipole responses in terms of wave‐vector‐dependent resonance frequency *ω*_0_(**k**) [17]. *ω*_0_(**k**) involves two dominant terms of *k* ^2^ by exciton's center‐of‐mass motion and *k* ^1^ by long‐range electron‐hole exchange, where *k* = |**k**|. In the low‐wavevector limit on the order of the free‐space wave number, these **k**‐dependence terms yield small *ω*_0_ shift on the order of 10^2^ GHz, which is three orders of magnitude smaller than *ω*_0_ in the order of 10^2^ THz [17]. Therefore, the local electromagnetic model in our approach provides a valid approximation in the exciton‐resonance bands, as previously reported in photonic waveguiding demonstrations with TMDC monolayers [1, 2, 5]. However, the spatial dispersion of *ω*_0_(**k**) might become prominent for guided exciton–polariton resonances in TMDC monolayers because such resonances carry additional in‐plane wave vectors well beyond the light line [18]. In such cases, nonlocal electromagnetism beyond our approach here should be introduced for proper description.

Likewise, plasmon polaritons in doped graphene carry large in‐plane wave vectors near the surface‐plasmon frequency [19, 20], and the local sheet model becomes invalid if their wave‐vectors are comparable to the Fermi wave vector set by the doping level [19, 21]. At typical doping levels with *E*_*F*_ ≈ 0.4–1 eV, the Fermi wave vector is on the order of 10^9^ m^−1^ [21]. In the visible and infrared spectral regions, near‐wavelength period photonic lattices provide wave vectors in the order of 10^6^ m^−1^, which is sufficiently smaller than the Fermi wave vector and the local‐conductivity sheet model should be valid for proper theoretical descriptions.

### 2.2 Horizontal Guided‐Mode Resonance

A zero‐OPL *δ*‐dielectric sheet produces robust elementary optical responses of partial reflection and waveguiding as discussed in the previous section. We further investigate two exemplary cases where the elementary responses are coherently combined and enable remarkably strong optical effects.

The first case is horizontal arrays of zero‐OPL *δ*‐dielectric sheets as a sort of simplest promising structures for potential applications. We consider a subwavelength‐periodic array structure, which is schematically illustrated in Figure 2A. The structure is defined by polarizing‐strength coefficient *L*_*δ*_ of the sheet segments, segment width *w*, and period *Λ*. In such a structure, the partial reflection at the sheet segments periodically modulates an incident wave and, thereby, produces multiple diffracted wavelets at discrete angular orders. Guided‐mode resonance (GMR) that strongly changes intensity and phase of the reflection and transmission emerges as a sharp spectral feature at a phase‐matching condition—one of the diffraction orders matches its in‐plane wavevector (*k*_*z*_) to the effective propagation constant of a guided mode [22, 23, 24, 25, 26, 27].

**FIGURE 2:**
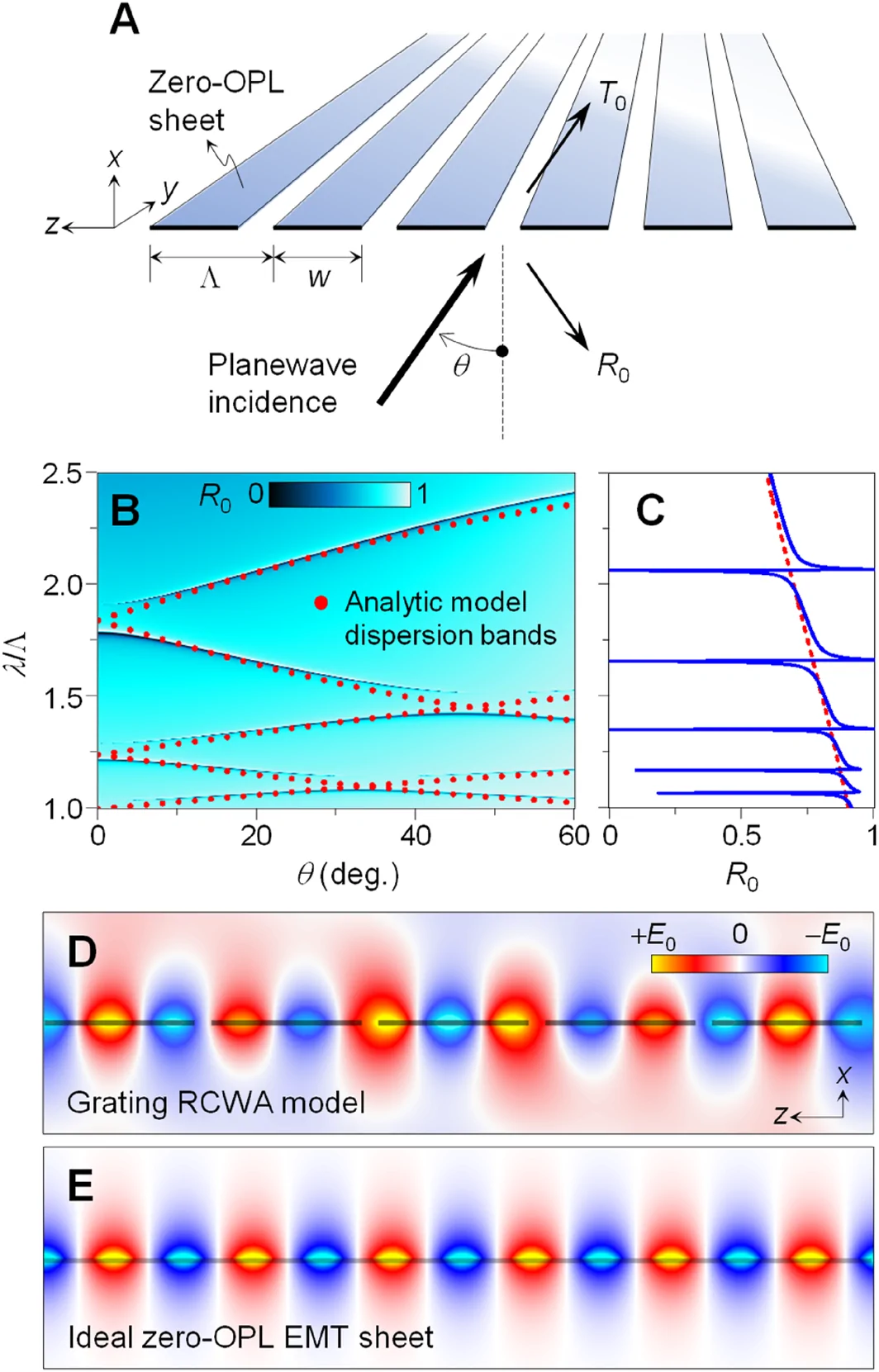
Horizontal guided‐mode resonance. (A) Schematic of a horizontal array of zero‐OPL *δ*‐dielectric sheets. (B) Reflectance map of an approximate‐slab model grating (*Λ* = 1.5 μm, *w* = 1.35 μm, *ε*_*s*_ = 1 + 10^4^, *h* = 0.15 nm) as a function of incidence angle *θ* and normalized wavelength *λ*/*Λ*. The overlaid red dots indicate analytic model dispersion bands due to Equations ((11), (12), (13), (14)) for the fundamental guided mode supported in an ideal zero‐OPL EMT sheet (*L*_*δ*_ = 1.35 μm). (C) Spectral reflectance profile (blue solid curve) taken from (B) at *θ* = 20° in comparison with the nonresonant reflectance spectrum (red dotted curve) of an ideal zero‐OPL EMT sheet due to Equations ((8), (9), (10)). (D) Electric field (*E*_*y*_) distribution at the resonance center at *λ*/*Λ* = 1.66 and *θ* = 20° in the RCWA model. (E) Guided‐mode *E*_*y*_ distribution for the ideal zero‐OPL EMT sheet at *λ*/*Λ* = 1.66.

In this scenario, the approximate GMR dispersion band can be conveniently calculated in terms of unit‐cell phase delays $\Delta \phi_{\text{GM}}$ for the guided‐mode field and $\Delta \phi_{\pm 1}$ for the ±1‐order diffracted field. In our case here, they are given by

(13) $$\Delta \phi_{\pm 1}\left(\theta ,\lambda \right)=n_{b}k_{0}\Lambda \;\sin\;\theta \pm 2\pi ,$$

(14) $$\Delta \phi_{\text{GM}}\left(\lambda \right)=n_{f}\left(\lambda \right)\;k_{0}\Lambda .$$

The GMR dispersion relation is obtained by their simple matching as

(15) $$\Delta \phi_{\text{GM}}\left(\lambda \right)=\Delta \phi_{\pm 1}\left(\theta ,\lambda \right).$$

We confirm our theoretical prediction by comparing it with numerical simulation based on the rigorous coupled‐wave analysis (RCWA) [28, 29, 30, 31]. Unfortunately, the ideal Dirac‐*δ* function in *ε* cannot be treated by the standard RCWA approach. Therefore, we employ an approximate model—a thin high‐index dielectric slab following Equation (3) in the deep‐subwavelength OPL‐thickness limit of *ε*_*s*_^1/2^ h≪λ. In Supporting Information S1: Section S1, we confirm that, in a deep‐subwavelength thickness limit of *h*/*λ* < 10^−2^, this approximate‐slab model precisely simulates the propagation constant and wavefunction of the ideal case‐guided mode from Equation (11).

The predicted GMR clearly appears in our sufficiently converged RCWA model, as shown in Figure 2B for the angular spectrum map and Figure 2C for the spectral profile at the angle of incidence *θ* = 20°. See the figure caption for detailed parameter conditions. The resonance profiles show typical GMR features such as asymmetric Fano‐resonance line shape and a frequency gap at *θ* = 0°.

The simulated spectrum from the RCWA model is consistent with our theoretical prediction. In Figure 2B, we indicate the dispersion curves due to Equations (10), (11), (13), and (14). They properly describe the GMR loci. In the theoretical curve calculation, we adopt the effective‐medium theory (EMT) for taking proper polarizing‐strength coefficient *L*_*δ*_ in the inhomogeneous grating layer. For a given segment‐width fill factor *F*_*w*_ = *w*/Λ, we evaluate EMT dielectric constant $\varepsilon_{s}^{\text{EMT}}=F_{w}\varepsilon_{s}+\left(1-F_{w}\right)\varepsilon_{b}$ and take EMT polarizing‐strength constant $L_{\delta }^{\text{EMT}}=\left(\varepsilon_{s}^{\text{EMT}}-1\right)h$ for the corresponding zero‐OPL *δ*‐dielectric sheet. In Figure 2D,E, we compare electric‐field distributions for the guided mode on the *δ*‐dielectric sheet and for the RCWA‐simulated GMR at *λ*/*Λ* = 1.66. It again confirms that the GMR in the RCWA model is essentially resulting from the guided mode in the ideal zero‐OPL *δ*‐dielectric sheet.

### 2.3 Vertical Guided‐Mode Resonance

Another exemplary case of strong optical responses is a vertical array structure, as schematically illustrated in Figure 3A. The optical field incident on such a structure is periodically modulated primarily by the first‐order‐derivative discontinuity footprint. Although this is in contrast to the horizontal arrays where the spatial field modulation originates from the uneven distribution of the partial reflection, the periodically modulated field anyway implies that the structure produces a significant diffraction effect. The periodically modulated incident field results in excitation of Bloch–Floquet modes within the array. Therefore, if one or multiple Bloch–Floquet modes have modal impedances significantly mismatching to the background medium, they may create lateral GMRs and Fabry–Pérot‐like resonances due to high internal reflection, which might enable versatile spectral features in the zero‐order fields, as is the case for high‐index GMR structures [32].

**FIGURE 3:**
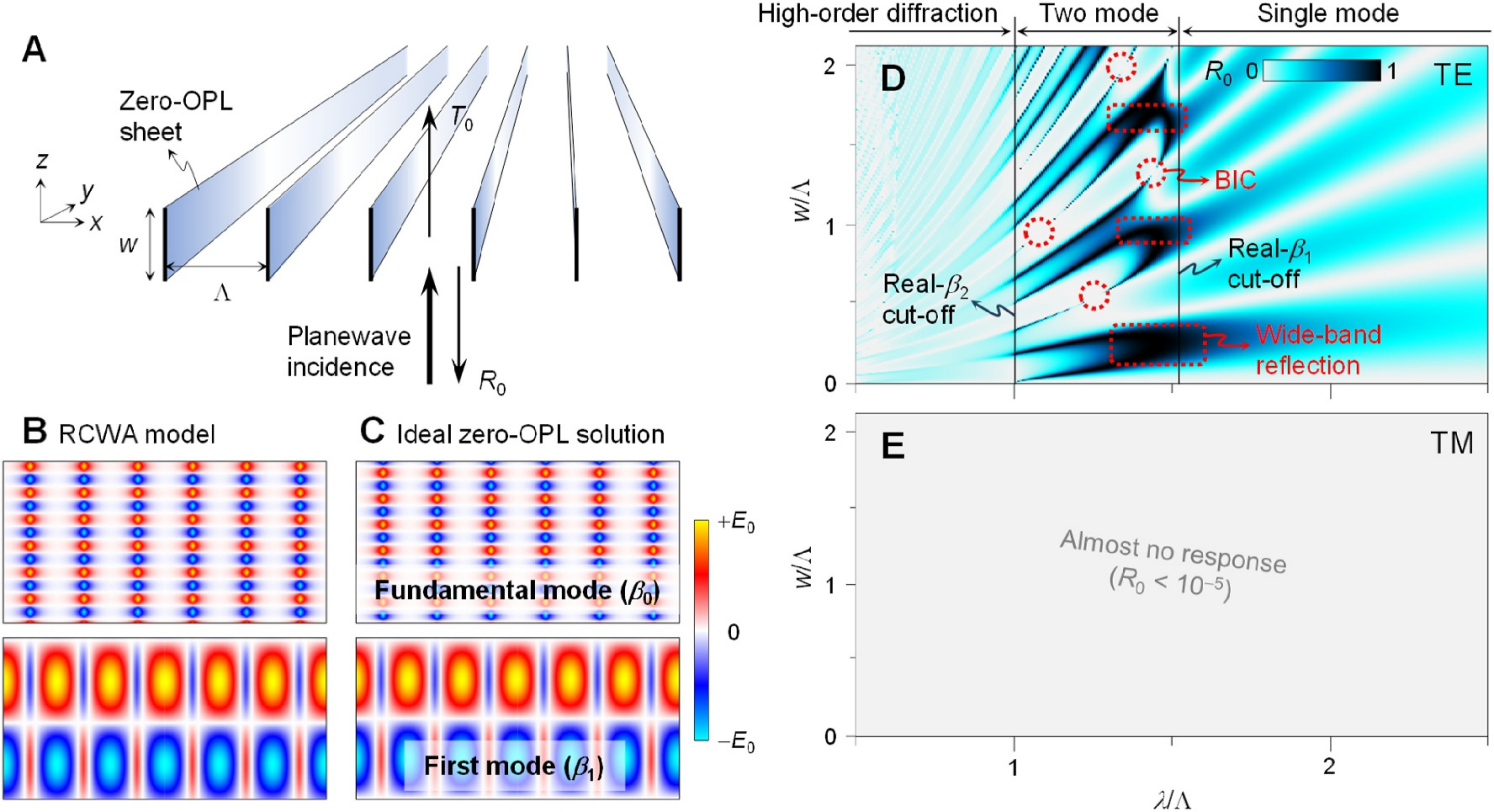
Vertical guided‐mode resonance. (A) Schematic of the vertical array of zero‐OPL *δ*‐dielectric sheets. Electric field distribution of (B) Bloch–Floquet modes for the approximate RCWA model (*Λ* = 1.5 μm, *ε*_*s*_ = 1+10^2^, *h* = 15 nm) in comparison with (C) ideal zero‐OPL *δ*‐dielectric sheet array (*L*_*δ*_ = 1.5 μm) at *λ*/*Λ* = 1.467 and *θ* = 0°. Reflectance maps of the vertical array of the approximate RCWA model as a function of normalized wavelength *λ*/*Λ* and normalized width *w*/*Λ* for (D) TE polarization and (E) TM polarization. In (D), useful resonance features are indicated with red‐dotted boxes for wide‐band high‐reflection plateaus and circles for bound‐states in the continuum. Note in (E) that the TM reflectance is almost constant at 0 within 10^−5^ range.

Bloch–Floquet mode solutions in the vertical array structure are readily found by applying the Bloch–Floquet theorem combined with the boundary conditions in Equations (5), (6), (7). The resulting dispersion relation for TE polarization is

(16) $$\cos\left(k\Lambda \right)=\cos\left(\alpha_{\nu }\Lambda \right)-\sigma_{\delta }\;\sin\left(\alpha_{\nu }\Lambda \right),$$

where *ν* is a mode‐number integer, $\alpha_{\nu }\equiv {\left(\varepsilon_{b}k_{0}^{2}-\beta_{\nu }^{2}\right)}^{1/2}$, with *β*_*ν*_ being the propagation constant along the *z*‐axis, which is the unknown to be determined by this dispersion relation for a given Bloch wavevector *k* along the *x*‐axis and vacuum wavenumber *k*_0_. Note that solutions with purely real‐valued *β*_*ν*_ potentially produce resonant responses. See Supporting Information S1: Section S2 for details of mathematical treatment leading to Equation (16) as well as complete wavefunction expressions of the Bloch–Floquet modes. For the sake of convenience, we assign mode number *ν* ascending from 0 such that $\text{Re}\left(\beta_{\nu }\right)>\text{Re}\left(\beta_{\nu +1}\right)$ for propagating (purely real *β* solution) Bloch–Floquet modes, whereas $\text{Im}\left(\beta_{\nu }\right)<\text{Im}\left(\beta_{\nu +1}\right)$ for evanescent (purely imaginary *β* solution) Bloch–Floquet modes.

We confirm the HCG analogy of our suggested vertical array structure by numerical analyses based on the approximate RCWA model used in the previous section. For a given thickness fill factor *F*_*h*_ = *h*/Λ, the approximate model follows Equation (3) such that (*ε*_*s*_−1)*F*_*h*_ = *L*_*δ*_/Λ = *constant* and we perform RCWA calculations in a very small fill‐factor limit of $F_{h}\ll 1$. In Figure 3B,C, we compare the Bloch–Floquet‐mode solution from Equation (16) with the approximate RCWA model for the case for *L*_*δ*_/*Λ* = 1. Other parameters are provided in the figure caption. We confirm that the approximate RCWA model converges well to the solution for the ideal zero‐OPL *δ*‐dielectric sheet array in both propagation constant *β* and field distribution in a small fill‐factor limit of *F*_*h*_ below 10^−2^.

Finally, we calculate reflectance spectra for vertically truncated arrays as a potentially useful resonant structure. In Figure 3D,E, we show the reflectance spectra (*R*_*s*_ = |*r*_*s*_|^2^ for TE polarization and *R*_*p*_ = |*r*_*p*_|^2^ for TM polarization) as functions of vacuum wavelength *λ* and sheet width *w* for a sufficiently converged model at *F*_*h*_ = 10^−2^. The *R*_*s*_(*λ*, *w*) map for TE polarization in Figure 3D shows remarkably useful resonance features including wideband reflection and bound‐states in the continuum as indicated therein. We note that the useful spectral features appear between the vertical demarcation lines, indicating the real *β*_1_ and *β*_2_ cut‐off wavelengths obtained from Equation (16) for the ideal zero‐OPL case. In this region, two propagating Bloch–Floquet modes (*β*_0_ and *β*_1_) are supported in the structure and their *w*‐dependent interference produces the effects which make the structure useful for a variety of applications. In the short wavelength region below the real‐*β*_2_ cut‐off, the lattice produces high‐order diffraction in the background media and subsequently the efficient zero‐order wave effects are no longer available.

All these characteristics are in common with conventional 3D‐medium high‐contrast GMR structures [32]. In such structures, strong resonance features are produced by the excitation of *β*_1_ mode in which the first‐order harmonic component is dominant. As the first‐order harmonic component is an evanescent order in the background media, this mode leads to a long‐lived confinement within the lattice region and thereby provides a key resonance mechanism. The wide‐band reflection and BIC features in Figure 3D are produced on this basis. In particular, the BIC features in our case here are the vertical cancellation types whose frequency and wave‐vector points are continuously tunable primarily with *w* change [33]. See Supporting Information S1: Section S3 for their spectral and field‐pattern properties. In contrast, the wide‐band reflection features are caused by constructive inter‐modal coupling between *β*_0_ and *β*_1_ modes. In contrast to *β*_1_ mode, *β*_0_ mode has the zero‐order harmonic as its dominant field component and produces weak and broad Fabry–Pérot resonance features. The wide‐band reflection features are created by simultaneous excitation of these two modes and their constructive interference at the reflection‐side boundary of the lattice [32].

The *R*_*p*_(*λ*, *w*) map for TM polarization in Figure 3E does not have any remarkable spectral features in stark contrast. The map appears as a light‐gray monotone density, indicating *R*_*p*_(*λ*, *w*) < 10^−5^ throughout the entire region of our calculation. This is understood by our explanation in the physical basis section. Under normal incidence of TM polarized light, **E**_*s*_^‖^ = 0 in the incident field and the sheets are thereby invisible to the incident field. This TM transparency, however, gradually degrades as the structure departs from the ideal zero‐OPL limit, as shown in Supporting Information S1: Section S4. We note that this extreme polarization selectivity is exactly the same as in the previous experimental demonstration for high‐aspect‐ratio Si grating structures a decade ago [34].

## 3 Conclusion

In conclusion, we have shown that a polarizing sheet with zero‐OPL thickness is the minimal embodiment of optical effects and can enable remarkably useful optical properties by appropriately structuring it to support resonant excitations. We have proposed an intuitive model and fundamental theory for elementary optical responses of partial reflection and waveguiding effects in the ideal zero‐OPL‐thickness case. Using rigorous numerical analyses, we have confirmed basic properties of the ideal‐case theory and remarkably strong optical responses from horizontal and vertical arrays of zero‐OPL‐thickness sheets due to excitations of guided modes and Bloch–Floquet modes in the same manner as their three‐dimensional structure counterparts [12, 13, 14, 15, 16, 17, 22].

Intriguingly, our result suggests a conceptual ground for creating minimally space‐occupying, most flexible, and least inertial optical or electromagnetic components based on 2D materials or any strongly polarizing media. For example, extremely high *ε*_*s*_ in the order of 10^2^–10^3^ in the MHz–GHz region is available in some ceramics including CaCu_3_Ti_4_O_12_, Cu_2_Ta_4_O_12_, and La_15/8_Sr_1/8_NiO_4_ [8, 9, 10, 11]. Taking the approximation scheme in Equation (3) into account, one may construct compact‐volume and light‐weight components from media with *σ*_*δ*_ ∼ 1 and physical thickness (*h*) in the order of 10^−3^–10^−4^ *λ*. Even in the infrared spectral domain, we note that optically transparent semiconductors such as Si and Ge have dielectric constant ∼ 10–20. They can produce a versatile resonance spectrum in a remarkably sparse grating condition with *F*_*h*_ ∼ 0.1, as previously demonstrated [23].

In another consideration, polarizing‐strength coefficients *L*_*δ*_ (or *σ*_*δ*_) and subsequent elementary properties in Equations ((8), (9), (10), (11)) can be efficiently used for experimentally treating optical properties of extremely thin material systems such as 2D materials [1, 2, 3, 4, 5, 6, 7] and nm‐thick island films [35, 36, 37]. In such systems, electric permittivity is poorly defined as a macroscopic optical parameter in principle and so do their experimental determination by conventional methods based on reflection intensity and phase measurement. In contrast, *L*_*δ*_ or *σ*_*δ*_ can be precisely determined by the conventional measurement methods with Equations ((8), (9), (10)) because they are strictly local in a normal axis while being macroscopic in the in‐plane dimension by their physical definitions—surface dipole density under the influence of the unit electric field. Therefore, they should be more reasonable to be used for optical property analyses than the conventional permittivity‐based approaches.

We note that our approach here can be further developed for high‐dimensional cases of polarizing wires and points, that is, $\chi \left(r\right)=L_{\delta }^{2}\;\delta \left(x,y\right)$ or $L_{\delta }^{3}\;\delta \left(x,y,z\right)$. In such cases, approximating real material systems can be found by the following conditions in analogy to Equation (3) as

(17) $$\varepsilon =1+L_{\delta }^{2}\;a^{-1}\;\text{or}\;1+L_{\delta }^{3}\;V^{-1},$$

where *a* is the cross‐sectional area of a wire and *V* is the volume of a pointy object in deep‐subwavelength critical dimensions. It is of our great interest for further in‐depth study in pursuing certain strong responses from their periodic or quasi‐periodic arrays, as they might enable novel optical or electromagnetic devices taking advantages of further enhanced extreme material sparsity.

## 4 Methods

All numerical simulation results presented in this work were obtained using a commercial rigorous coupled‐wave analysis package (MC Grating software) based on the collinear method. The number of Fourier modes was chosen according to the convergence behavior of the Fourier coefficients, which depends on the fill factor. Reflectance spectra in Figure 2B,C were calculated with 31 space harmonics, whereas those in Figure 3D,E used 131 harmonics to ensure convergence for an extremely small fill factor at 0.01. The electric‐field distribution in Figure 2D was computed using the structural parameters specified in the figure caption. In Figure 3B for the Bloch modes, the infinitely thick array structure is simulated with a finite‐thickness array with an impedance‐matched absorbing layer at the bottom. The total field from this calculation is then spatial‐frequency‐filtered to extract pure Bloch modes.

## Author Contributions

**Da Yong Lee:** methodology, investigation, validation, formal analysis, visualization, writing – original draft, writing – review and editing. **Moongul Byun:** methodology, validation, investigation, formal analysis, visualization, writing – original draft, writing – review and editing. **Robert Magnusson:** conceptualization, investigation, writing – original draft, writing – review and editing, validation, formal analysis, methodology. **Jae Woong Yoon:** conceptualization, methodology, software, data curation, investigation, validation, supervision, funding acquisition, project administration, resources, writing – original draft, writing – review and editing.

## Funding

This research was supported by the Leader Researcher Program (NRF‐2019R1A3B2068083) from the National Research Foundation of Korea.

## Conflicts of Interest

The authors declare no conflicts of interest.

## Supporting information


Supporting Information S1

## Data Availability

The data that support the findings of this study are available from the corresponding author upon reasonable request.
