# Supplementary material for: Optical Responses From Zero Optical‐Path‐Length Object
Source: Nanophotonics. 2026 Aug 10;15(15):e70243. doi: 10.1002/nap2.70243 (PMC13642499; doi:10.1002/nap2.70243)
Supplement: Supplementary file 1 [file NAP2-15-e70243-s001.pdf]

Supporting Information for
Optical responses from zero optical-path-length objects

Da Yong Lee,[1, †] Moongul Byun,[1, †] Robert Magnusson,[2] and Jae Woong Yoon[1,*]

[1]Department of Physics, Hanyang University, Seoul 133-791, Korea
[2]Department of Electrical Engineering, University of Texas – Arlington, TX 76019, United States

S1. Convergence of the thin high-index-film model to a zero-OPL δ-dielectric sheet

In horizontal array structure, we adopt the effective-medium theory in grating layer, as described in the main text. The periodic layer is treated as a homogeneous medium with dielectric constant $\varepsilon_s^{EMT} = F_w\varepsilon_s + (1-F_w)\varepsilon_b$. For the corresponding δ-dielectric sheet, the EMT polarizing-strength coefficient is given by $L_\delta^{EMT} = (\varepsilon_s^{EMT}-1)h$, and its effective propagation constant, denoted as $\beta_\delta = k_0 n_f$, can be obtained using Equation (11) in the main text. In a thin high-index dielectric slab case, the effective propagation constant, denoted as β_s, is obtained from the standard dispersion relation for the fundamental TE mode of a symmetric dielectric slab waveguide

$$h\sqrt{k_0^2\varepsilon_s^{EMT} - \beta_s^2} = \tan^{-1}\left(\frac{\sqrt{\beta_s^2 - k_0^2\varepsilon_b}}{\sqrt{k_0^2\varepsilon_s^{EMT} - \beta_s^2}}\right) \tag{S1}$$

For a consistent comparison between the δ-dielectric sheet and a thin high-index dielectric slab, the surface dipole density is kept constant. Consequently, reducing h necessarily increases $\varepsilon_s^{EMT} = 1 + L_\delta^{EMT}/h$. We compare the β_δ and β_s by varying the normalized slab thickness h/Λ (or h/λ). As show in Figure S1, for the given structural parameters, β_s approaches β_δ as h/Λ (or h/λ) goes to zero, confirming convergence of the slab-model solutions to the δ-dielectric-sheet ones.

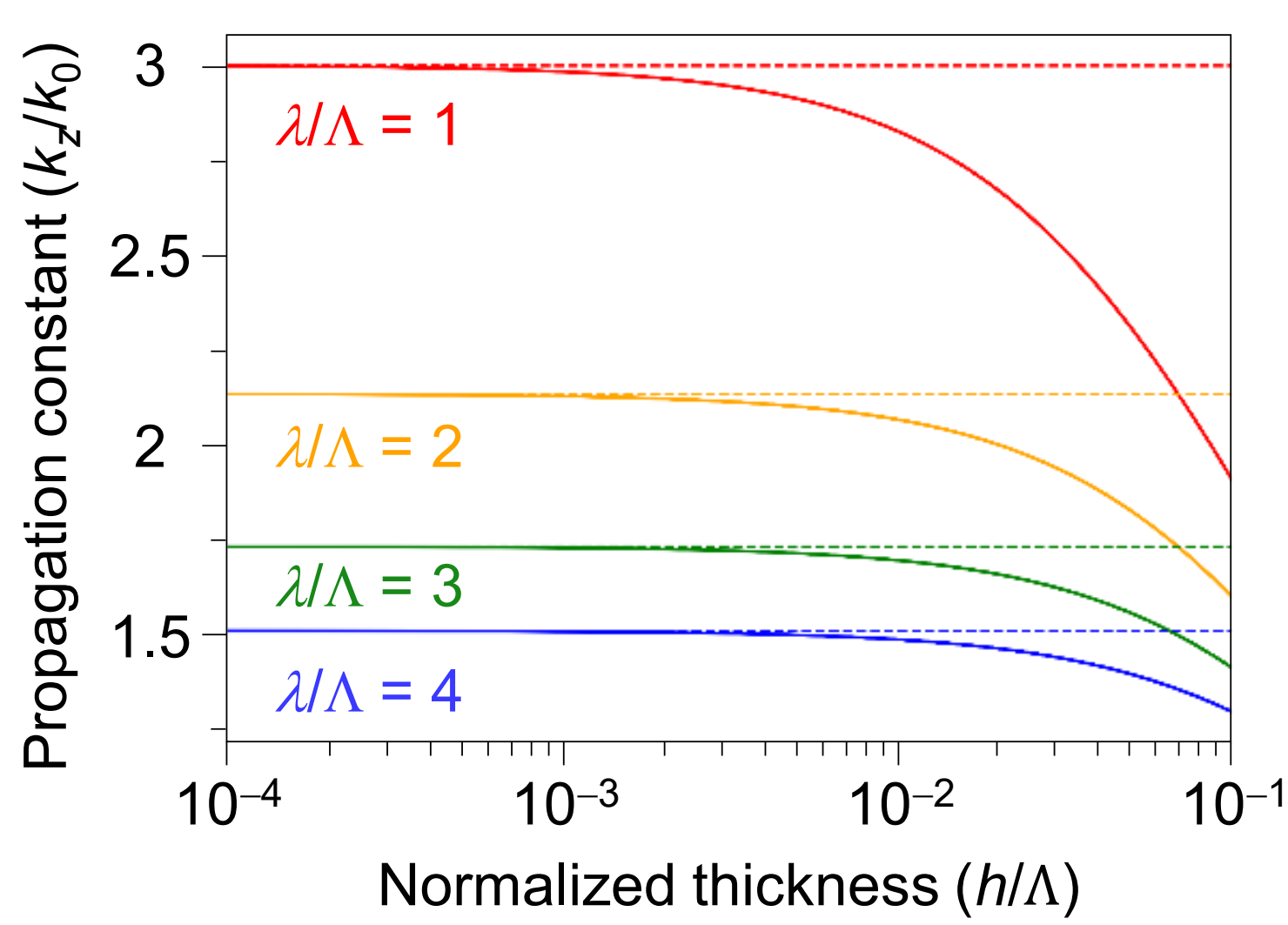

Figure S1. Comparison of propagation constants between δ-dielectric sheet and thin high-index-film model. Real propagation constants of the waveguide mode along the z-axis from δ-dielectric sheet (dotted lines, with L_δ^{EMT} = 1.35 μm) and thin high-index dielectric slab with EMT dielectric constant ε_s^{EMT} (solid lines) for normalized wavelengths λ/Λ = 1, 1.5, 2 and 2.5, plotted as a function of normalized slab thickness h/Λ. The normalization is taken with respect to the lattice period Λ = 1.5 μm. The δ-sheet corresponds to a fixed EMT polarizing-strength coefficient, while the dielectric slab varies with h_{slab}, demonstrating convergence of β_s to β_δ in the deep-subwavelength thickness limit.

S2. Exact Bloch-Floquet mode solution for a vertical array of zero-OPL δ-dielectric sheets

In this note, we derive the dispersion relation for the TE-polarized field in a vertical array structure, corresponding to Equation (16) in the main text. We consider an array with period Λ and a polarizing-strength coefficient L_δ. Within the j-th unit cell ($j \in \mathbb{Z}$), the ansatz for the x-component of the electric field **E** is given by

$$E_j(x) = A_j e^{i\alpha_v x} + B_j e^{-i\alpha_v x} \tag{S2}$$

where α is defined in the main text. We now impose the boundary conditions at $x = \mathrm{p}\Lambda$ as given in Equation (6) and Equation (7) of the main text, relating $E_\mathrm{p}(x)$ and $E_{\mathrm{p}+1}(x)$ across the δ-interface. This yields

$$A_j + B_j = A_{j+1}e^{-i\alpha_v\Lambda} + B_{j+1}e^{i\alpha_v\Lambda} = \frac{i}{2\sigma_\delta}\left[(A_j - B_j) - (A_{j+1}e^{-i\alpha_v\Lambda} - B_{j+1}e^{i\alpha_v\Lambda})\right] \tag{S3}$$

where σ_δ is defined in the main. We now apply the Bloch-Floquet theorem, $E_{\mathrm{p}+1}(x+\Lambda) = E_\mathrm{p}(x)e^{ik\Lambda}$, to Equation (S1). This implies the following recursion relations:

$$A_{j+1} = A_j e^{ik\Lambda} \tag{S4}$$

$$B_{j+1} = B_j e^{ik\Lambda} \tag{S5}$$

If we apply these relations into Equation (S2), we obtain two independent relations between $\underline{A}_\mathrm{p}$ and B_p, such that

$$\frac{A_j}{B_j} = -\frac{e^{i\alpha_v\Lambda/2}\sin\left[(k+\alpha_v)\Lambda/2\right]}{e^{-i\alpha_v\Lambda/2}\sin\left[(k-\alpha_v)\Lambda/2\right]} = \frac{e^{i\alpha_v\Lambda/2}\sin\left[(k+\alpha_v)\Lambda/2\right] + \sigma_\delta e^{-ik\Lambda/2}}{e^{-i\alpha_v\Lambda/2}\sin\left[(k-\alpha_v)\Lambda/2\right] - \sigma_\delta e^{-ik\Lambda/2}} \tag{S6}$$

By solving this equation, we get the dispersion relation Equation (16) in the main text. Once the structural information is given, we can solve the propagation constant along z-axis, $\beta_v = (\varepsilon_\mathrm{b}k_0^2 - \alpha_v^2)^{1/2}$. We compare the real-$\beta$ solutions from this dispersion relation and the counterparts in high-contrast grating (HCG) by varying its fill factor. See Figure S2 for this comparison with the structural information. In this figure, note that the β solutions of HCG converge to those of exact δ-dielectric-sheets case under the decrease of fill factor.

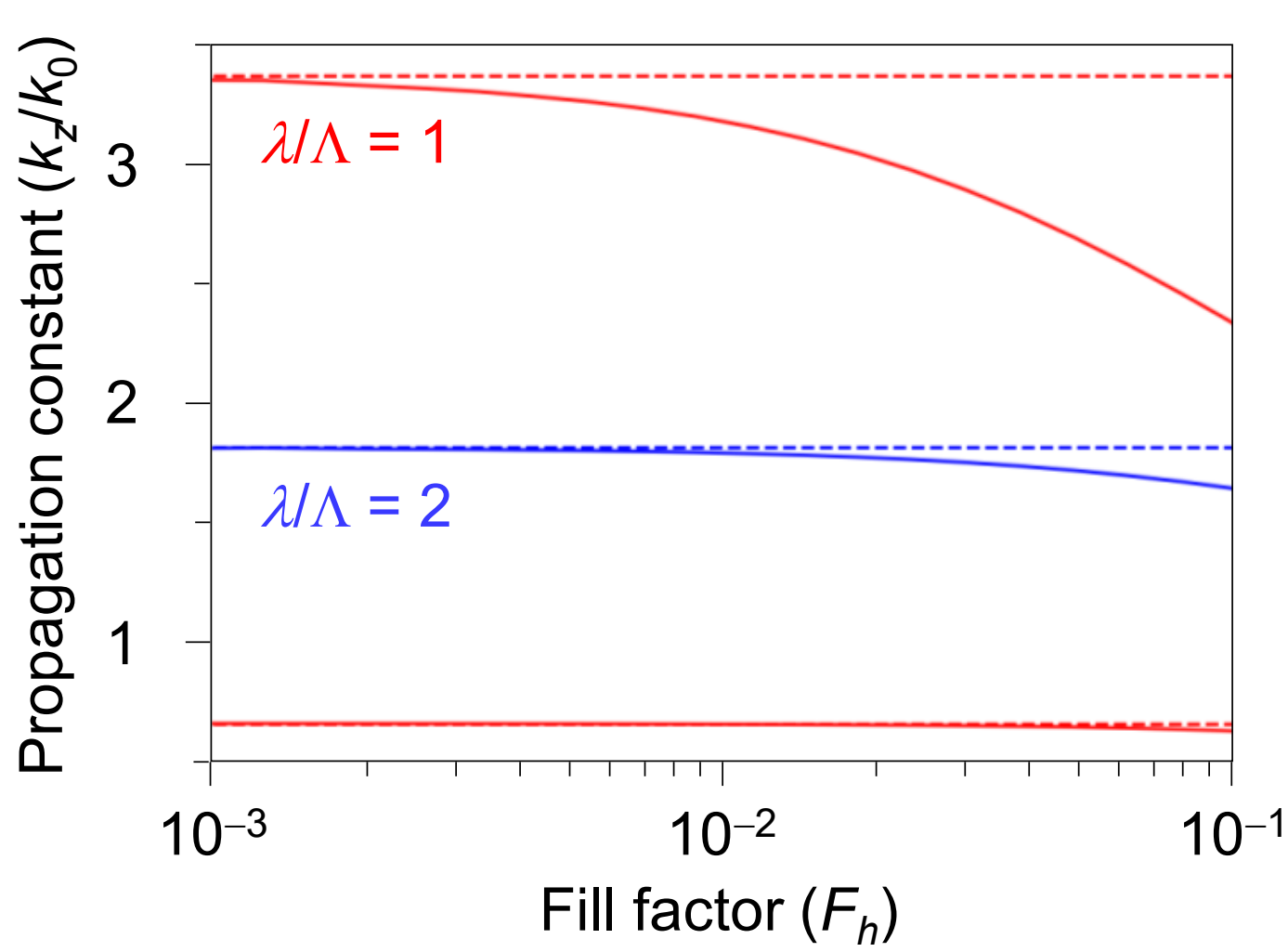

Figure S2. Comparison of propagation constants between δ-dielectric sheet and high-contrast grating. Real propagation constants along the z-axis from the exact δ-dielectric-sheets dispersion (dotted lines) and its high-contrast grating (HCG) counterpart (solid lines) for normalized wavelengths λ/Λ = 1 and 2, plotted as a function of the HCG fill factor F_h. The grating parameters are $\Lambda = L_\delta$ = 1.5µm and (ε_s -1)F_h = 1. This comparison demonstrates the convergence of β in HCG to β_δ in the small fill-factor limit.

S3. Angle-resolved spectral properties of BICs in vertical arrays

In this section, we identify the BIC type for the features revealed in the TE reflectance map in Figure 3D of the main text. The four BIC features in Figure 3D have common characteristics as a parameter-tuned, vertical cancellation BIC type in their spectral, angular, and field-pattern properties. We select the BIC feature at $\lambda/\Lambda = 1.44$ and $w/\Lambda = 1.34$ as a representative case. This feature is marked with the red-dotted circle in Figure S3(a). The field pattern in the inset shows relative electric field at this BIC condition. Obviously, this bound state is not the symmetry-protected type with its mode field having laterally even-symmetry with respect to the structural mirror-symmetry plane. Instead of the symmetry protection, its formation mechanism is the vertical cancellation of the leakage-radiation field as reported by Peng et al. in their foundational paper [33] of the main text. According to [33], a vertical-cancellation BIC is supported by complete destructive interference of leakage-radiation wavelets from the vertically decomposed lattice components. It does not require strong coupling between multiple resonance states as required for Friedrich-Wintgen BICs nor strict odd-symmetry in the lateral wave function as required for symmetry-protected BICs. Therefore, frequency and wave-vector point for a vertical-cancellation BIC is continuously tunable within the corresponding resonance band under lattice height or period change. This continuous tunability is a signature characteristic of a vertical-cancellation BIC and clearly confirmed in the BIC features in Figure 3D, as summarized in Figure S3.

Figures S3(b) and S3(c) show w-λ maps of the reflectance for off-Γ-point conditions at $\theta = \pm 8°$ and $\pm 28°$. All BIC features for the Γ-point condition in Figure 3D or equivalently Figure S3(a) continuously change their wavelengths without any extinction as a signature as vertical-cancellation BICs. In Figures S3(d)–S3(f), we show θ-λ maps of the reflectance to reveal properties of the selected representative BIC in the resonance bands. As lattice height w increases from 1.34Λ to 1.70Λ, it continuously increases its angular and spectral point to larger θ and larger λ along the corresponding resonance band. We note that there is no off-Γ band crossing associated with this BIC feature, confirming that no Friedrich-Wintgen-type mechanism is involved in this BIC formation.

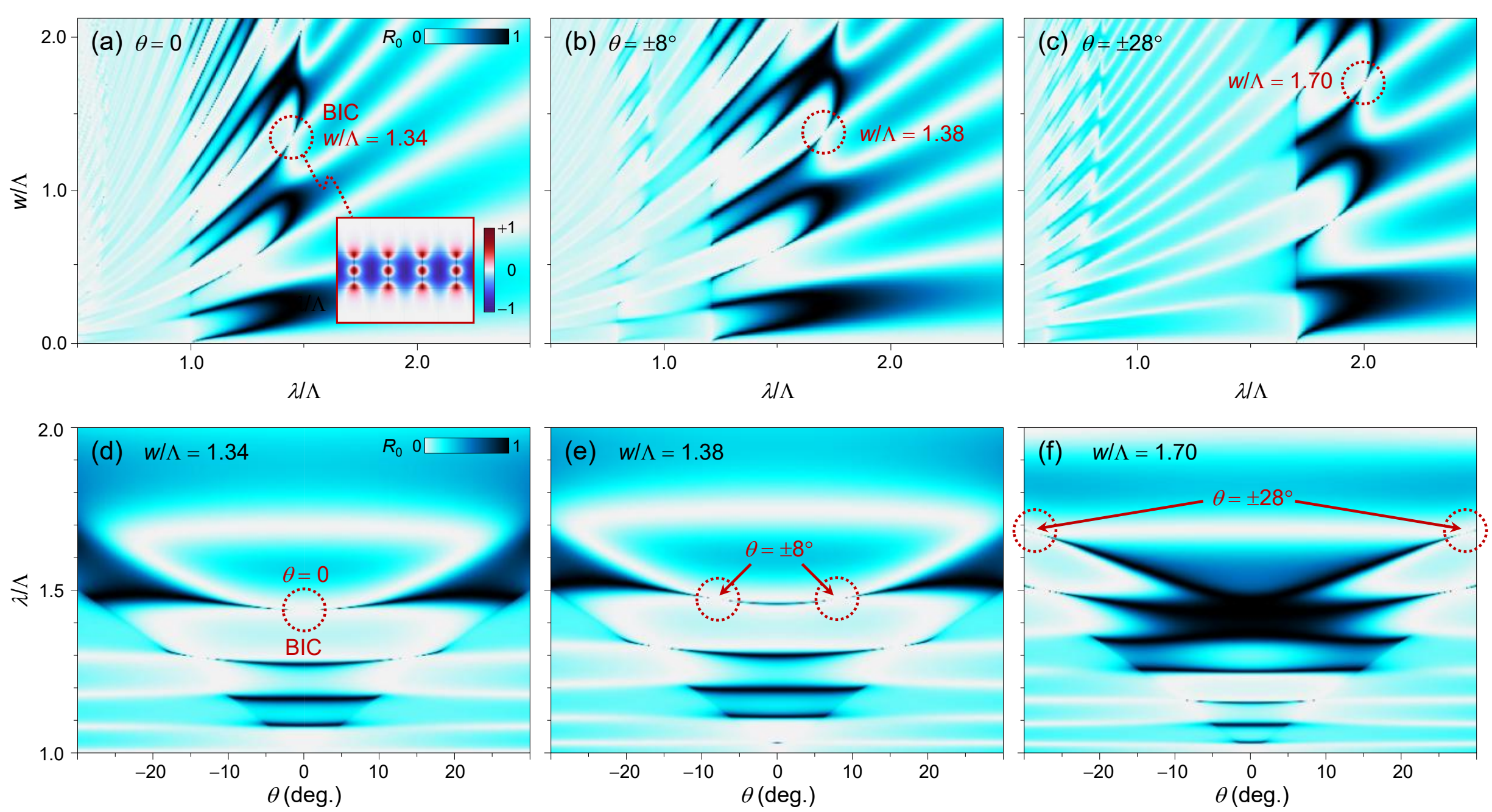

Figure S3. Array-height (w) dependence of the reflectance spectra for different angles of incidence at (a) $\theta = 0$, (b) $\theta = \pm 8°$, and (c) $\theta = \pm 28°$. Inset field pattern in (a) shows snapshot of relative electric-field distribution at the BIC point marked with the red-dotted circle. Angle-resolved reflectance spectra for different array heights at (d) $w/\Lambda = 1.34$, (e) $w/\Lambda = 1.38$, and (f) (d) $w/\Lambda = 1.70$. These spectra show gradual change of spectral and angular location of the BIC marked with a red-dotted circle, which is identified as a tunable, vertical-cancellation BIC.

S4. Transparency to normally incident TM-polarized light

In the ideal zero-OPL case, a sheet responds only to the tangential component of the electric field, $\mathbf{E}_s^{//}$, as described by the boundary conditions in Equation (6) and Equation (7) in the main text. This behavior can be understood from the finite-thickness slab that we use as the fictitious medium in Equation (3). At the slab interface the tangential electric field is continuous, whereas the normal electric field is discontinuous, as required by continuity of the normal electric displacement. The normal electric field inside the slab, $E_i^{\perp}$, is therefore suppressed relative to the surrounding background, $E_o^{\perp}$, by a factor $1/\varepsilon_s$, i.e. $E_i^{\perp} = E_o^{\perp}/\varepsilon_s$ for a vaccum background (n_b=1). The surface polarization can be expressed by $P_s^{\perp} = \varepsilon_0(\varepsilon_s-1)E_i^{\perp}h = \varepsilon_0(1-\varepsilon_s^{-1})E_o^{\perp}h$. In the vanishing-thickness limit $h \to 0$ taken at fixed $L_\delta = (\varepsilon_s-1)h$, the normal surface polarization is linearly proportional to h and eventually vanishes. For the tangential component, by contrast, continuity of the tangential electric field dose not impose the suppression of electric field in the slab, so that the resulting tangential surface polarization $P_s^{//} = \varepsilon_0(\varepsilon_s-1)E_i^{//}h = \varepsilon_0 L_\delta E_i^{//}$ comes out independent of h and remains finite. An ideal zero-OPL sheet therefore responds only to the tangential electric field. This is fully consistent with the spectrally featureless normally incident TM reflectance map in Figure. 3E and the absence of a fundamental TM guided mode on a single sheet, as described by Equation (12).

For a slab with non-zero OPL thickness, however, the amount of normal surface polarization remains finite. Therefore, this slab is no longer completely transparent to the normally incident TM-polarized light whose electric field is entirely normal to the sheet. Figure S4(a) shows the real-β Bloch-mode solutions of the vertical approximate RCWA as a function of the fill factor. Among the modes, the fundamental mode confined to the slab exists without a cutoff and dominates the spectral features of the normal incidence TM response. As the fill factor decreases, the mode confined to the slab, which has $\beta/k_0 > 1$ at finite thickness, progressively delocalizes and its propagation constant converges to k_0. This can be viewed as the vanishing-thickness slab gradually losing the strength of its normal surface polarization.

Figure S4(b) and Figure S4(c) present the normal incidence TM reflectance for fill factors of 0.1 and 0.075, respectively. Both values lie away from the deep-subwavelength thickness regime, so that the dispersion has not yet fully converged, in contrast to the smaller fill factor of 0.01 used for the converged map in Figure 3E. At a fill factor of 0.1, the response is nearly transparent over most of the zero-order region but still exhibits weak resonance lines, indicating a small optical effect at finite thickness. As the fill factor is reduced to 0.075, these resonances weaken further and the reflectance approaches the spectrally featureless behavior of Figure 3(e). Taken together, these results indicate that the vertical approximate RCWA model recovers the transparency of the ideal zero-OPL array to normally incident TM-polarized light in the limit of a sufficiently small fill factor, where the normal surface polarization is suppressed and, correspondingly, the dispersion of the confined mode converges to k_0.

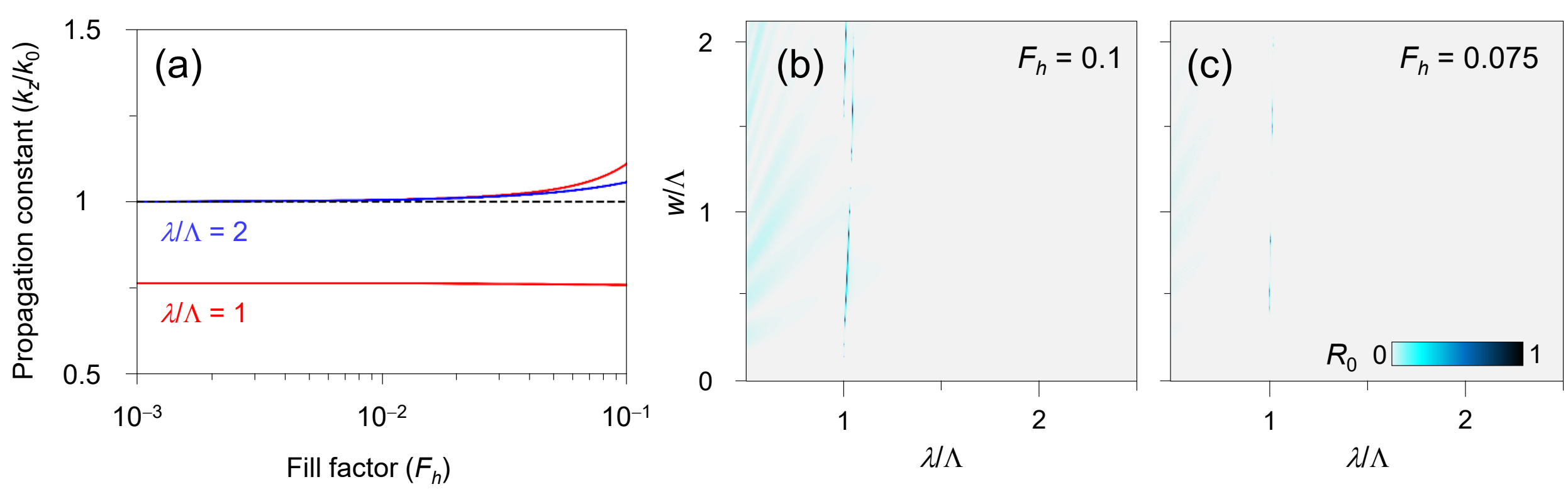

Figure S4. (a) Surface-normal propagation constants of real-β TM Bloch-Floquet modes as functions of fill factor F_h at two selected wavelength values. Zero-order reflectance map on a wavelength-sheet-thickness plane for (b) $F_h = 0.1$ and (c) $F_h = 0.075$.
